# Deficits in reinforcement learning but no link to apathy in patients with schizophrenia

**DOI:** 10.1038/srep40352

**Published:** 2017-01-10

**Authors:** Matthias N. Hartmann-Riemer, Steffen Aschenbrenner, Magdalena Bossert, Celina Westermann, Erich Seifritz, Philippe N. Tobler, Matthias Weisbrod, Stefan Kaiser

**Affiliations:** 1 Department of Psychiatry, Psychotherapy, and Psychosomatics; Psychiatric Hospital; University of Zurich, Switzerland; 2 Psychiatric Hospital Karlsbad Langensteinbach, Karlsbad, Germany; 3 Laboratory for Social and Neural Systems Research, Department of Economics, University of Zurich, Zurich, Switzerland; 4 University of Heidelberg, Heidelberg, Germany

## Abstract

Negative symptoms in schizophrenia have been linked to selective reinforcement learning deficits in the context of gains combined with intact loss-avoidance learning. Fundamental mechanisms of reinforcement learning and choice are prediction error signaling and the precise representation of reward value for future decisions. It is unclear which of these mechanisms contribute to the impairments in learning from positive outcomes observed in schizophrenia. A recent study suggested that patients with severe apathy symptoms show deficits in the representation of expected value. Considering the fundamental relevance for the understanding of these symptoms, we aimed to assess the stability of these findings across studies. Sixty-four patients with schizophrenia and 19 healthy control participants performed a probabilistic reward learning task. They had to associate stimuli with gain or loss-avoidance. In a transfer phase participants indicated valuation of the previously learned stimuli by choosing among them. Patients demonstrated an overall impairment in learning compared to healthy controls. No effects of apathy symptoms on task indices were observed. However, patients with schizophrenia learned better in the context of loss-avoidance than in the context of gain. Earlier findings were thus partially replicated. Further studies are needed to clarify the mechanistic link between negative symptoms and reinforcement learning.

Schizophrenia is a debilitating psychiatric disorder that is characterized by positive symptoms (hallucinations and delusions), negative symptoms (anhedonia, avolition, asociality, blunted affect, and alogia), and cognitive deficits. A consensus has been established, that negative symptoms can be grouped into two separable dimensions^1^,^2^. First, a dimension comprising anhedonia, avolition, and asociality. In line with previous studies^3^,^4^,^5^, we refer to this dimension as apathy. Second, diminished expression, consisting of blunted affect and alogia. Negative symptoms are mostly resistant to pharmacological treatment^6^ and at the same time critical for functional outcome^7^,^8^,^9^. Consequently, the recent decade has seen an increase in research efforts to understand mechanisms underlying negative symptoms in schizophrenia. A prominent line of research has implicated that negative symptoms might reflect disordered processing of rewards and value-based decision-making^10^,^11^. In particular, these concepts are relevant for the negative symptom dimension apathy, which reflects a reduction in motivation and goal-directed behavior^12^.

Two fundamental processes can be distinguished in value-based decision-making: prediction errors (PEs) and the representation of the expected outcome value^10^. Prediction errors serve as a learning signal at the time of an outcome. Positive PEs code for better than expected outcomes, while negative PEs code for worse than expected outcomes. Positive PEs have been associated with increased phasic firing rates of dopamine neurons in the midbrain, whereas negative PEs have been linked to brief cessations of firing in these neurons^13^. It is assumed that positive PEs reinforce current motor responses and associations by dopaminergic projections mainly to the striatum and frontal corteIn contrast, when a negative PE is elicited through a worse than expected outcome, previously learned associations are weakened. A second mechanism crucially important for value-based decision-making regards the representation of outcome values. At the time of choice, a subject has to compare expected outcome values of different choice options (situation-action pairs), which rely on precise representations of these previously learned values. The orbitofrontal cortex (OFC) is thought to be significantly involved in the representation of expected value^14^. Thus, value-based decision-making relies on a learning mechanism at the time of outcome, which relates to relevant previous situations (PE signaling in the midbrain) and a “storage unit” that holds a representation of the expected value of choice options (expected value representation in the OFC).

Selective reinforcement learning deficits with reduced learning from positive, but intact learning from negative outcomes has been reported in patients with schizophrenia^15^,^16^,^17^, which is in line with a proposed neurocomputational model of dopamine induced basal ganglia-cortex interactions^15^,^18^. It has further been argued that this selective deficit might be linked to the severity of negative symptoms^16^,^19^. The selective learning deficit in the context of reward constitutes a plausible factor causing and/or maintaining negative symptoms, especially motivational deficits manifesting themselves as apathy. However, it is unclear whether these findings reflect impairments in positive PE signaling during outcomes or in the precise representation of expected reward values to guide decision-making. In their seminal paper, Gold and colleagues^20^ applied a task that allowed the investigation of the relative contribution of these two mechanisms. In an acquisition phase, participants were presented with stimulus pairs involving either potential gain or potential loss. Following this acquisition phase, where participants learned the stimulus-outcome associations of these pairs, they indicated the valuation of the different stimuli in a transfer test phase. In this transfer test phase, participants chose between novel combinations of all learned stimuli without additional feedback. The most critical novel stimulus combination was the one that involved choosing between an action that had been rewarded and one that avoided loss. Both these stimuli should have elicited positive PEs during acquisition and thus should have been reinforced. If an actor would only make decisions based on PE signals during learning, he would be indifferent between the rewarded and the loss-avoiding stimulus. In contrast, if the actor would base his decision on representations of expected value, he should prefer the rewarded to the loss-avoiding stimulus, since the latter does not yield reward. Patients with high apathy symptoms showed impairments in learning from gain but intact loss-avoidance learning. Moreover, patients with high apathy levels failed to prefer the rewarded stimuli (that with higher expected value) to the loss-avoiding stimuli in the transfer test phase, indicating deficits in the representation of expected value.

The findings of Gold and colleagues^20^ have provided new insights and contributed to a mechanistic understanding of motivational negative symptoms in schizophrenia. In light of the relevance of this pattern and the neurobiological plausibility thereof, we aimed to assess the stability of the finding across studies as part of a larger study investigating neural and cognitive correlates of negatives symptoms in schizophrenia. Based on the study by Gold and colleagues^20^ and other previous studies^15^,^16^,^17^, we hypothesized that patients compared to healthy control participants would show impaired gain, but intact loss-avoidance learning in the acquisition phase. Moreover, we expected that patients would generally show better learning in loss-avoidance relative to gain-seeking trials, as revealed by within-group analyses. Critically, we hypothesized that both these effects would be more pronounced in patients with high apathy compared to patients with low apathy symptoms. Moreover, based on the paper of Gold and colleagues^20^, we expected that in the transfer test phase high apathy patients, compared to low apathy patients and controls, would show a weaker preference for rewarded stimuli over the stimuli that simply avoided loss, which would be indicative of impaired expected value representation.

## Methods

### Participants

Participants included 64 patients with a DSM-IV^21^ diagnosis of schizophrenia (*n* = 60) or schizoaffective disorder (*n* = 4; no mood episode; the patient group is abbreviated SZ), recruited from the in- and outpatient units of the Psychiatric University Hospital in Zurich (Switzerland) and the Psychiatric Hospital Karlsbad Langensteinbach (Germany). Patients were stable regarding medication and psychopathology and met the following inclusion criteria: Daily lorazepam dosage ≤1 mg, no florid positive symptoms (Positive and Negative Syndrome Scale; PANSS^22^ no positive item score >4), no extrapyramidal symptoms on clinical examination, no additional DSM-IV axis I or II diagnosis. Nineteen healthy control participants (HC) were recruited from the hospital staff of the Psychiatric University Hospital in Zurich. In order to confirm axis I diagnosis in the SZ group, and exclude potential HC group participants with axis I disorders, we conducted the Mini-International Neuropsychiatric Interview^23^.

Participants did not enter data analysis if learning performance in the probabilistic learning task did not exceed 0.5. In other words, they had to perform better than would be expected in case of choice by chance. 7 participants from the SZ group and 1 HC participant failed to reach the criterion and were thus excluded from further analyses. All remaining patients received antipsychotic medication. Eight were also treated with antidepressants, 5 with mood stabilizers, 1 with an anxiolytic agent, and 2 with anticholinergic medication. Further clinical and demographic information of the study sample are presented in Table 1.

The ethics committee of the Canton of Zurich approved the study protocol and all methods and experimental procedures were performed in accordance with the latest version of the Declaration of Helsinki. All participants provided written informed consent.

### Assessment of psychopathology and cognition

To assess the symptom severity in our patient sample, we applied the PANSS^22^. To further assess negative symptoms more accurately and according to the recent consensus regarding their two-dimensionality (i.e., apathy and diminished expression)^1^,^2^, we used the German version of the Brief Negative Symptom Scale (BNSS)^24^. Patients were divided into a high apathy (HA) and a low apathy (LA) group according to a median split on the apathy factor (anhedonia, asociality, and avolition) of the BNSS. Please note that Gold and colleagues^20^ applied the Scale for the Assessment of Negative Symptoms^25^ and extracted a factor comprising avolition and anhedonia global items^26^, which is similar to the apathy factor of the BNSS^27^. Thus the study by Gold and colleagues and the present study have targeted the same psychopathological construct. Social, occupational, and psychological functioning of patients were evaluated with the Global Assessment of Functioning scale (GAF)^28^. Participants were also characterized regarding premorbid intelligence using a multiple-choice vocabulary intelligence test^29^. We further applied the digit-symbol coding test of the Wechsler Intelligence Scale^30^, which assesses processing speed and has been shown to significantly predict composite cognition scores in schizophrenia^31^.

Patients differed from the HC group regarding processing speed and premorbid verbal intelligence (see Table 1). Groups did not significantly differ regarding age and gender. The LA and HA group showed differences in gender distribution (relatively more men in the HA group), in total negative symptom severity, in severity of positive symptoms (more severe positive symptoms in the HA group), global psychopathology (more severe global symptoms in the HA group), and in functioning (lower functioning in the HA group).

### Probabilistic reward learning task

The present learning task is adapted from the version introduced by Gold and colleagues^20^. In a pilot study, we implemented the task using images of landscapes as learning stimuli as in the original Gold study. However, five patients with schizophrenia all failed to learn stimulus-outcome associations above chance level. Consequently, we adjusted the task in several respects. First, we replaced the landscape stimuli with line drawings of everyday objects (taken from a larger set of 260 pictures by Snodgrass and Vanderwart)^32^. Second, to emphasize the learning context (gain vs. loss-avoidance), we added green or red bars above and below the stimuli. Third, instead of the verbal feedback in the trials with “zero outcome” (“Not a winner, Try again!” or “Keep your money!”), we presented an empty white circle. Each of these adaptions was aimed at making the task easier for the participants.

**Figure 1:**
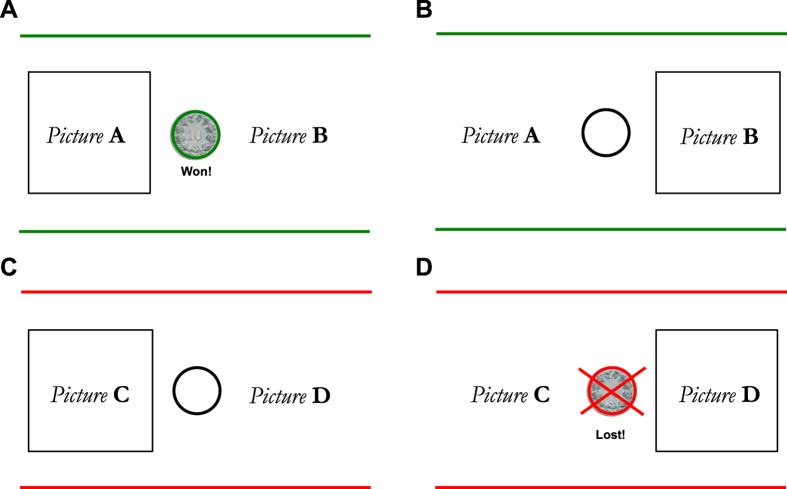
Reinforcement learning task during the acquisition phase. Participants were presented with 2 reward and 2 loss-avoidance pairs in a total of 160 learning trials. Half of the pairs were reinforced in 90% of the time, while the other half was reinforced in 80% of the time. (**A**) Feedback after a correct choice in the reward-seeking trials. (**B**) Feedback delivered following an incorrect choice in the reward trials. (**C**) Feedback after correct choice in the loss-avoidance trials. (**D**) Feedback delivered following an incorrect choice in the loss-avoidance trials. Picture stimuli were selected from a larger set of line drawings by Snodgrass and Vanderwart^32^.

The resulting adapted version of the task was administered via the MATLAB toolboxes *Cogent 2000* and *Cogent Graphics* on a 19-in monitor. The task is structured in two phases, an acquisition and a transfer phase. During the acquisition phase, participants were presented with 4 pairs of line drawings of everyday objects (white on black background), 1 pair at a time (see Fig. 1). Two pairs involved a potential gain if the correct item was selected and two pairs involved a potential loss if the incorrect item was selected. For the gain pairs, if the correct item was selected, participants saw the image of either 10 Swiss cents (≈0.096 US dollars by time of testing) or 10 Euro cents (≈0.090 US dollars by time of testing) coupled with the feedback “Win!” (positive PE), whereas if the incorrect item was selected, an empty white circle was presented, indicating that nothing was won in this trial (“zero outcome”, negative PE). The correct response was reinforced 90% of trials in one pair and 80% in the other pair. Two other pairs involved a potential loss (loss-avoidance pairs). In these pairs, selection of the incorrect response resulted in the feedback “Lose!” combined with a crossed out image of the coin (negative PE). If the correct item was selected in the loss-avoidance pairs, participants avoided a loss in 90% or 80% of the time and were presented with an empty white circle (“zero outcome”, positive PE). A 12-trial practice session was administered to ensure task comprehension, followed by 160 learning trials, which were presented in randomized order within 4 blocks. Each pair was presented 40 times in the acquisition phase.

Following acquisition, the 64 trials of the transfer test phase were presented. The original pairings of the acquisition phase were each presented 4 times, and 24 novel pairings were each presented twice. Novel pairings consisted of each trained item combined with one another (e.g., a 90% winner item was paired with both items from the 80% gain pair, the 90% loss-avoidance pair, and the 80% loss-avoidance pair). No feedback was given during the transfer test phase. Participants were instructed to select the item that they thought was “best” based on prior learning in the acquisition phase.

### Statistical analyses

To investigate performance in the acquisition phase, we computed a repeated-measures analysis of variance (ANOVA) with the between-subject factor group (HC, LA, HA) and within-subjects factors of feedback valence (gain vs. loss-avoidance), probability (80% and 90%), and learning block (1–4). Huynh-Feldt correction was applied if assumption of sphericity was violated. Significant interaction terms were followed-up by post hoc Fisher’s Least Significant Difference (LSD) contrasts. In line with Gold and colleagues^20^, we subtracted performance in loss-avoidance trials from the performance in gain-seeking trials (gain-loss difference score) to additionally investigate whether learning was better in the context of gain or loss-avoidance. Potential group differences were then examined using ANOVA, while within-group balance between gain vs. loss-avoidance learning was examined using one-sample t-tests. Performance in the transfer test phase was evaluated using ANOVA, follow-up post hoc LSD tests, and one-sample t-tests to test for within-group preferences between stimuli.

Please note that *p*-values of one-sample t-tests were not corrected for multiple testing. Raw task data was processed using MATLAB. All statistical analyses were performed using SPSS version 22.0.

## Results

### Acquisition phase

**Figure 2:**
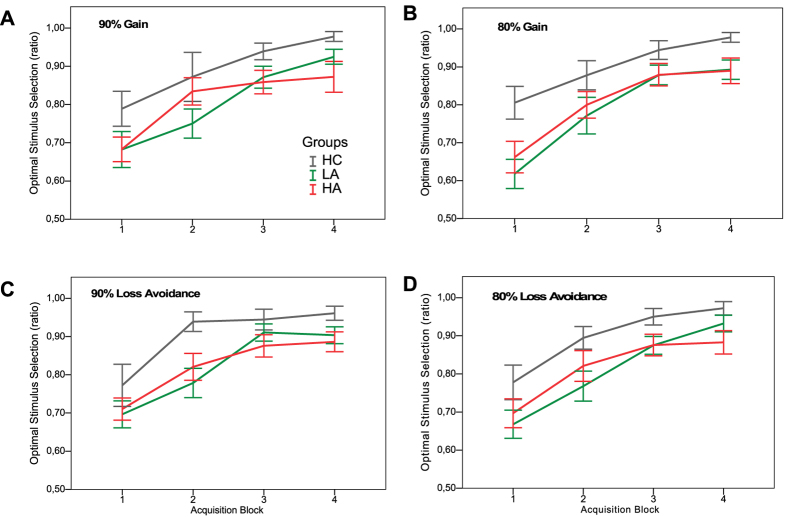
Acquisition phase. Learning in the 90% (A/C) and 80% (B/D) probability gain (A/B) and loss-avoidance trials (C/D). HC = Healthy control group. LA = Low apathy symptom group. HA = High apathy symptom group. Error bars indicate SEM.

As depicted in Fig. 2, all groups showed learning across the 4 blocks in all conditions of the acquisition phase. However, the HC group performed better than the patient groups in all conditions, while the LA and the HA groups did not show consistent differences in learning performance. More precisely, a mixed-effects ANOVA with the factors group, feedback valence, probability and learning block revealed significant main effects of group (*F*(2,72) = 4.01, *p* = 0.02) and learning block (*F*(3,72) = 81.20, *p* < 0.001). Follow-up LSD tests yielded significant group differences between the HC and the LA (*p* = 0.01) and HA (*p* = 0.03), but no significant difference between the two patient groups (*p* = 0.80). The significant main effect of learning block reflects significant learning performance across blocks. All other main effects, as well as interaction effects (two-way and three-way) were non-significant.

**Figure 3:**
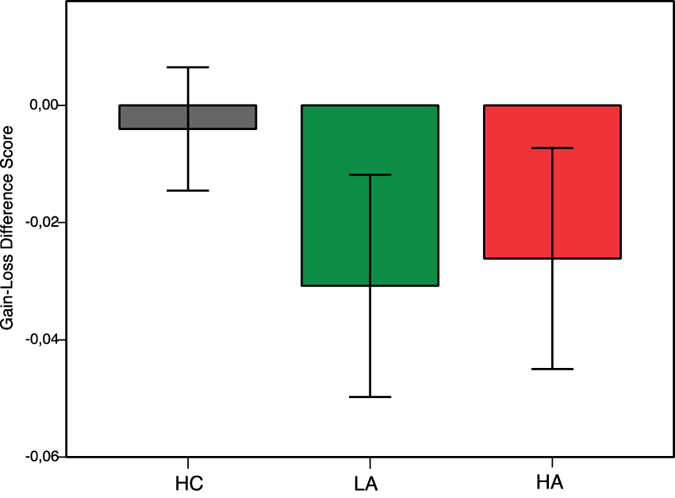
Performance on the gain-loss difference score among patients and healthy controls. Above zero translates to better gain-seeking learning as compared to loss-avoidance learning (below zero the other way around). HC = Healthy control group. LA = Low apathy symptom group. HA = High apathy symptom group. Error bars indicate SEM.

In line with Gold and colleagues^20^, we additionally calculated a gain-loss difference score to investigate the balance of learning from gain vs. loss-avoidance (Fig. 3). We subtracted learning performance in loss-avoidance trials from the learning in gain trials. An ANOVA with this gain-loss difference score as dependent variable yielded no significant group differences between HC, LA, and HA groups (*F*(2,72) = 0.51, *p* = 0.60). One-sample t-tests were conducted to investigate the balance of gain vs. loss-avoidance learning within the groups. The HC group did not show a preference for feedback valence in the learning phase (*t*(17) = −0.38, *p* = 0.71). The patient groups showed a tendency towards better loss-avoidance than reward learning, however, the effects were not significant (LA: *t*(27) = −1.62, *p* = 0.12 ; HA: *t*(28) = −1.39, *p* = 0.18). We further pooled the two patient groups to explore potential effects disregarding negative symptom severity. The pooled patient group showed significantly better learning in the context of loss-avoidance than in context of reward (*t*(56) = −2.14, *p* = 0.04).

In sum, in the acquisition phase all groups showed learning across blocks. The HC group performed significantly better than the patient groups, which did not differ from each other regarding acquisition learning. Moreover, the pooled patient group learned better in the context of loss-avoidance relative to gain trials independent of apathy symptom severity.

### Transfer test phase

**Figure 4:**
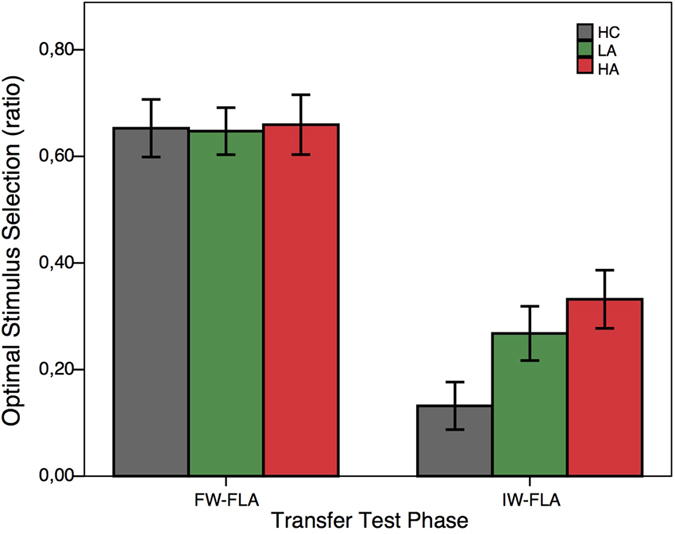
Transfer test phase. Bars on the left depict the percentage of optimal choice (i.e., the one with higher expected value) when faced with the frequent winner (FW) and frequent loss-avoiding (FLA) item. Bars on the right refer to the infrequent winner (IW) and frequent loss-avoiding pair (FLA). HC = Healthy control group. LA = Low apathy symptom group. HA = High apathy symptom group. EV = Expected value. Error bars indicate SEM.

In the transfer test phase (Fig. 4), participants were presented with novel stimulus pairings and were instructed to choose the stimulus that they thought was “best” based on prior learning.

Two stimulus pairings in the transfer test phase are indicative of the relative contribution of PEs and representations of expected value. A first relevant pair is the one with the most frequently rewarded stimulus (FW) and the item that most frequently avoided losses (FLA). Both stimuli were associated with identical positive PEs during acquisition learning. However, the expected value of the FW stimulus (frequently wins 10 centimes) is higher than the FLA item (frequently avoids the loss of 10 centimes). The one-way ANOVA testing for this first critical group difference was not significant (*F*(2, 72) = 0.23, *p* = 0.79). One-sample t-tests on the FW-FLA pair, testing for difference from 0.5, revealed that the HC (*t*(17) = 2.83, *p* = 0.01), the LA (*t*(27) = 2.15, *p* = 0.04), and the HA group (*t*(28) = 4.48, p < 0.001) showed choice preference implicating, at least partial, representation of expected value. In other words, they preferred the stimulus with higher expected value, despite equal association with positive PEs.

The second relevant pairing in the transfer test phase was the pair with the infrequent winners (IW) and the frequent loss-avoiders (FLA). All participant groups showed a robust preference for the frequent loss avoiding items, although the infrequent winner items had a higher expected value (one-sample t-tests all *p*’s < 0.001). Thus, all groups preferred the stimulus that was more frequently associated with positive prediction errors to the stimulus that had a higher expected value, but was more frequently associated with negative prediction errors. The one-way ANOVA testing for group differences was only trend-level significant (*F*(2, 72) = 3.11, *p* = 0.051). In fact, patients showed a tendency for stronger preference for the higher expected value item compared to healthy controls.

In sum, there were no significant group differences in the transfer phase, which tested for learned valuation of the stimuli. Participants showed preference for the FW stimulus in the FW-FLA pairs, indicating expected value representation in all groups. In contrast, in the IW-FLA pairs, all groups preferred the stimuli with lower expected value but more frequent positive prediction errors, which suggests valuation that was more strongly driven by prediction errors. Thus, all groups showed a valuation pattern that is indicative of prediction error based learning combined with a representation of expected value.

### Effects of potential confounders

HC group performance in the cognitive measures used in the present study was significantly better compared to the SZ group. If cognitive measures were added as covariates in the reported mixed-effects ANOVA for the acquisition phase data, the main effect of group did not remain significant (*F*(2,70) = 0.05, *p* = 0.56). It can thus not be ruled out that differences in acquisition learning between the SZ and HC group were driven by general cognitive deficits in the SZ group. However, the gain-loss difference score was not significantly associated with cognitive measures in the study groups.

We additionally computed Pearson correlations coefficients (*r*) between positive symptom severity and chlorpromazine equivalents (CPZ) and relevant task indices. Interestingly, positive symptoms correlated negatively (*r* = −0.28, *p* = 0.04) with learning performance in the acquisition phase. Moreover, positive symptoms correlated negatively with the ratio of optimal selection in the FW-FLA pairs of the transfer phase (*r* = −0.30, *p* = 0.03), which might suggest that positive symptoms interfere with the ability to represent the expected reward value of actions. However, since the present study focused on negative symptoms, severe positive symptoms were an exclusion criterion to control for secondary negative symptoms. Thus positive symptom severity was low in the present patient sample. Please note that adding positive symptom severity as a covariate to the ANOVAs computed in the present paper had no significant influence on the results. CPZ equivalents correlated with the ratio of optimal selection in the FLA-IW pairs (*r* = 0.30, *p* = 0.02), which might indicate that antipsychotic medication may have a positive effect on reward value representation. CPZ equivalents and antipsychotic medication regimens did not differ significantly between the LA and HA group.

## Discussion

The present study aimed to investigate probabilistic reinforcement learning in the context of gain and loss-avoidance in a sample of schizophrenia patients and how learning and valuation relates to the severity of apathy. More specifically, we aimed to assess the stability of the findings of a previous study by Gold and colleagues^20^. We found a general deficit in probabilistic reinforcement learning in the patient group compared to healthy controls. However, LA and HA patients did not differ regarding acquisition learning performance. Moreover, we found that the pooled patient group learned better in the context of loss-avoidance relative to gain, independent of severity of apathy symptoms. In the transfer test phase, groups did not differ in their valuation in two critical stimulus pairs. In other words, diagnosis of schizophrenia and apathy symptom severity did not affect valuation. All participants showed choice behavior in the transfer test phase indicating both learning from positive PEs and representation of expected reward value. The present study could thus partially replicate the findings by Gold and colleagues^20^.

Several studies have suggested that patients with schizophrenia have a selective learning deficit when they have to learn contingencies of reward-related stimulus-outcome associations^15^,^16^,^17^,^19^,^20^. In the context of preventing a potential loss, learning performance seems to be unimpaired. This pattern has been shown to be most pronounced in patients suffering from severe negative symptoms^16^,^20^. An organism that is impaired in encoding and learning from positive outcomes and at the same time is unimpaired in learning from negative outcomes is likely to develop defensive strategies, and show decreased exploration and gain-oriented goal-directed behavior. This description matches the clinical symptoms of motivational negative symptoms (i.e., apathy) quite well. In the present study, we could replicate the relatively better learning from loss-avoidance compared to gain learning in patients with schizophrenia. However, both learning indices were impaired compared to the HC group, which is in contrast to some previous studies reporting intact loss-avoidance learning^15^,^16^,^17^,^19^,^20^, but in line with studies showing impairments in both gain and loss-avoidance learning in patients with schizophrenia^33^,^34^,^35^ and first episode psychosis^36^. In further contrast to some previous studies^16^,^19^,^20^, we found no association of apathy symptoms with a relative preference for loss-avoidance learning or other learning parameters. However, other recent studies have also reported no relation of learning parameters with apathy symptom severity^34^,^37^,^38^.

In the present study, we investigated choice in the transfer test phase in two critical stimulus parings. Contrary to our hypothesis, we did not find any significant group differences. One test pair (FW-FLA) indicated that all participant groups attained an expected value representation during acquisition. However, in the other critical pair (IW-FLA) all participant groups preferred the stimulus with lower expected value, but stronger association with positive prediction errors. These inconsistent findings likely reflect the fact that the proposed two relevant mechanisms (PEs and precise expected reward value representation) are not mutually exclusive, but rather dynamic systems that strongly interact. This notion is in line with the reasoning of Gold and colleagues^20^, which additionally supported the assumption of a “mixed-strategy” regarding the two mechanisms with elegant computational modeling. Considering non-significant group differences in transfer phase choice behavior, additional computational modeling is not indicated to address the primary hypotheses of the present study. In line with our findings, a recent study in patients with first episode psychosis has applied a very similar probabilistic reward learning task and also found no group differences between patients and healthy controls in transfer test performance^36^.

Certain limitations should be considered when interpreting the present findings. First, the sample size of the present study was modest and thus further studies are needed. Second, our patient sample included in- and outpatients. This approach was chosen to include a wide array of patients with different severity of negative symptoms. In the study by Gold and colleagues^20^, the patient sample was recruited from an outpatient clinic. It is thus possible that the heterogeneity in the patient group had an influence on the fact that task adaptations were needed to ensure comprehension and also consecutive results. Participants in the present study showed overall better learning in the acquisition phase compared to the original study. This difference might have also arisen because of the adaptions that were made. Moreover, it cannot be ruled out that the better learning in the acquisition phase impacted choice behavior in the critical transfer test phase. It is conceivable that an “overlearning” in the acquisition phase has led participant to develop a cognitive strategy to solely divide stimuli into “good” and “bad” items, which would decouple them from their feedback valence. The consistent preference in all groups for the stimulus with lower expected value in the IW-FLA pair would be in line with this reasoning. However, it could not explain the preference for the frequent winner over the frequent loss avoider (FW-FLA pair). Another possible factor that might explain discrepancies in our findings compared to the study by Gold and colleagues^20^ is the age difference in the two samples. Patients in our sample were on average more than 10 years younger, which might be associated with lower chronicity in our sample. As mentioned above, it has recently been reported that patients with first episode psychosis show less pronounced learning deficits and intact expected value representation^36^, which is more consistent with our findings and might point towards effects of illness stage on learning and value representation. A direct comparison of chronicity in our sample and the study by Gold and colleagues^20^ is not possible because they did not report illness duration. Our sample was also different regarding antipsychotic medication. Patients in our sample were only medicated with second-generation antipsychotics. However, a direct comparison of antipsychotic medication effects is not possible since Gold and colleagues^20^ did not report CPZ or haloperidol equivalents in their sample characteristics.

The present paper reports that schizophrenia patients showed a general deficit in probabilistic reinforcement learning and a tendency towards better loss-avoidance than gain learning. However, these effects were not driven by apathy symptom severity. These findings are in partial contrast to previous studies and thus add to the growing body of literature that reports heterogeneous findings regarding the link between task-based assessments and negative symptoms (e.g., effort-based decision-making). These inconsistencies might be due to critical variation in task design and characteristics of the study population. Future studies with unmedicated patients or studies applying functional magnetic resonance imaging might help to clarify the mechanistic link between negative symptoms and reinforcement learning and might also explain the heterogeneity in findings. Shedding light on these potentially basic mechanisms of schizophrenia symptoms is of fundamental importance not only for the development of pharmacological therapies, but also for psychosocial and psychotherapeutic interventions. For example, the success of cognitive behavioral therapy is strongly dependent on the patient’s ability to learn from experiences that contradict expectations (e.g., “better than expected” outcomes when challenging defeatist beliefs).

## Additional Information

**How to cite this article**: Hartmann-Riemer, M. N. *et al*. Deficits in reinforcement learning but no link to apathy in patients with schizophrenia. *Sci. Rep.* **7**, 40352; doi: 10.1038/srep40352 (2017).

**Publisher's note:** Springer Nature remains neutral with regard to jurisdictional claims in published maps and institutional affiliations.

## Figures and Tables

**Table 1:** Demographic and clinical information and cognitive test scores.

Characteristics	SZ (*n *= 57)	HC (*n* = 18)	*p (t, Χ*^*2*^)	LA (*n *= 28)	HA (*n* = 29)	*p (t, Χ*^*2*^)
Age	30.26 (8.59)	32.67 (6.93)	0.28	30.96 (7.72)	29.59 (9.44)	0.55
Gender (m/f)	40/17	13/5	0.87	12/16	5/24	0.04
Antipsychotic medication regimen						
Haloperidol or fluphenazine only	0	—	—	0	0	n.s.
Clozapine only	4	—	—	3	1	n.s.
Other second generation	24	—	—	11	13	n.s.
Clozapine plus another antipsychotic	8	—	—	3	5	n.s.
Other combination	21	—	—	11	10	n.s.
Other clinical characteristics						
CPZ equivalents	548.19 (410.79)	—	—	561.29 (432.19)	535.555 (396.28)	0.82
In-/outpatients	52/5			25/2	26/3	n.s.
Illness duration (months)	76.89 (77.24)	—	—	66.61 (59.78)	87.18 (91.43)	0.32
Age at illness onset	24.20 (7.57)	—	—	25.62 (8.36)	22.79 (6.54)	0.16
Number of previous hospitalizations	3.54 (2.35)	—	—	3.11 (2.06)	3.97 (2.56)	0.17
Negative symptoms (total score BNSS)	25.07 (12.58)	—	—	16.57 (8.07)	33.28 (10.57)	<0.001
Apathy (BNSS)	14.60 (7.87)	—	—	8.29 (4.43)	20.69 (5.18)	<0.001
Diminished expression (BNSS)	6.84 (5.36)	—	—	5.57 (3.90)	8.07 (6.29)	0.07
PANSS positive	10.75 (3.35)	—	—	9.50 (2.65)	11.97 (3.55)	0.004
PANSS negative	14.87 (5.71)	—	—	11.96 (3.26)	17.69 (6.18)	<0.001
PANSS global	25.26 (6.05)	—	—	23.18 (4.66)	27.28 (6.61)	0.009
GAF	54.37 (10.31)	—	—	59.43 (8.31)	49.48 (9.79)	<0.001
Cognition						
Processing speed (Digit-symbol-coding)	−1.72 (1.05)	0(0)	< 0.001	−1.70 (1.01)	−1.73 (1.11)	0.90
Premorbid verbal intelligence (MWT-B)	−0.90 (1.38)	0(0)	0.01	−1.00 (1.39)	−0.80 (1.39)	0.58

Cognitive test scores were z-transformed based on performance of the control group. SZ = Patient group. HC = Healthy control group. LA = Low apathy symptom group. HA = High apathy symptom group. CPZ = Chlorpromazine equivalents. BNSS = Brief Negative Symptom Scale. PANSS = Positive and Negative Syndrome Scale. GAF = Global Assessment of Functioning. MWT-B = Multiple-choice verbal intelligence test.
