# Corrigendum: Deficits in reinforcement learning but no link to apathy in patients with schizophrenia

**DOI:** 10.1038/srep44510

**Published:** 2017-04-11

**Authors:** Matthias N. Hartmann-Riemer, Steffen Aschenbrenner, Magdalena Bossert, Celina Westermann, Erich Seifritz, Philippe N. Tobler, Matthias Weisbrod, Stefan Kaiser

Scientific Reports 7: Article number: 4035210.1038/srep40352; published online: 01 10 2017; updated: 04 11 2017

In this Article, the affiliation for Matthias Weisbrod is incomplete. The correct affiliation is listed below:

Psychiatric Hospital Karlsbad Langensteinbach, Karlsbad, Germany.

Department of General Psychiatry, Center of Psychological Medicine, University of Heidelberg, Heidelberg, Germany.

